# Proprietary management and higher readmission rates: A correlation

**DOI:** 10.1371/journal.pone.0204272

**Published:** 2018-09-18

**Authors:** Manish Mittal, Chih-Hsiung E. Wang, Abigail H. Goben, Andrew D. Boyd

**Affiliations:** 1 Department of Pharmacology, College of Medicine, University of Illinois at Chicago, Chicago, Illinois, United States of America; 2 Department of Biomedical and Health Information Sciences, College of Applied Health Sciences, University of Illinois at Chicago, Chicago, Illinois, United States of America; 3 Library of the Health Sciences, University Library, University of Illinois at Chicago, Chicago, Illinois, United States of America; 4 Department of Medical Education, College of Medicine, University of Illinois at Chicago, Chicago, Illinois, United States of America; Medical University Graz, AUSTRIA

## Abstract

**Introduction:**

Reducing preventable readmissions among Medicare beneficiaries is an effective way to not only reduce the exorbitantly rising cost in healthcare but also as a measure to improve the quality of patient care. Many of the previous efforts in reducing readmission rate of patients have not been very successful because of ill-defined quality measures, improper data collection methods and lack of effective strategies based on data driven solutions.

**Methods:**

In this study, we analyzed the readmission data of patients for six major diseases including acute myocardial infarction (AMI), heart failure (HF), coronary artery bypass graft (CABG), pneumonia (PN), chronic obstructive pulmonary disease (COPD), and total hip arthroplasty and/or total knee arthroplasty (THA/TKA) from the Center for Medicare and Medicaid Readmissions Reduction Program (HRRP) program for the period 2012–2015 in context with the ownership structure of the hospitals.

**Results:**

Our analysis demonstrates that the readmission rates of patients were statistically higher in proprietary (for profit) hospitals compared to the government and non-profit hospitals which was independent of their geographical distribution across all six major diseases.

**Conclusion:**

This finding we believe has strong implications for policy makers to mitigate any potential risks in the quality of patient care arising from unintended revenue pressure in healthcare institutions.

## Introduction

Reducing preventable readmissions among Medicare patients has become an important national priority for healthcare policy makers. From the Medicare Payment Advisory Commission (MedPAC) report, almost one-fifth of Medicare beneficiaries discharged are readmitted within 30 days [1]. The majority of hospital readmissions fall in four big categories including: 1) postoperative surgical complications; 2) improper discharge care of patients; 3) reoccurrence of chronic conditions such as COPD or heart failure; and 4) patient’s error in medication adherence [2]. A 2014 report identified that 10–50% of all the readmissions are potentially preventable readmissions and cost Medicare $17.4 billion annually [3]. The heart failure and COPD patients have the highest readmission rate of 23%-26% among all the diseases [3].

Depending on the delivery of healthcare services, there are three main business models of providers including government, proprietary (for-profit), and non-profit [4]. The government healthcare institutions deliver healthcare services mostly from public funding, whereas the proprietary hospitals are owned by investors and shareholders who have self-financial interest and most of the profits from the proprietary hospital is distributed among them. The non-profit hospitals may be owned by the members of the organization, communities, regional health authorities, or a hospital trust. These owners do not derive any profits and the main purpose of non-profit hospitals is to provide healthcare services and maintain socio-economic stability within the organization. The non-profit hospital’s surplus profits may be invested in the research or teaching activities depending upon the goals and the mission of the organization. [4].

Previous studies in the literature have focused on the causative factors of patient readmission such as socio-economic factors or hospital-level care processes to reduce the readmissions rate [5–7]. The ownership structure of providers, however, has recently emerged as another crucial paradigm governing the readmission risk among hospitalized patients [4]. Notably, non-profit health care institutions have been shown to provide better quality of patient care than for-profit health care institutions in multiple studies [8–12]. Some studies, in contrary, have shown better patient outcomes in for-profit institutions [13,14]. However, little research has been done to report how the ownership structure of hospital operation impacts the readmission rates of the patients in different disease categories. In this study, we analyzed the readmission ratio from Hospital Readmission Reduction Program (HRRP) database for 2012–2015 for six major disease categories including acute myocardial infarction (AMI), heart failure (HF), coronary artery bypass graft (CABG), pneumonia (PN), chronic obstructive pulmonary disease (COPD), and total hip arthroplasty and/or total knee arthroplasty (THA/TKA), in context of the ownership structure of the providers nationwide.

## Methods

**Fig 1:**
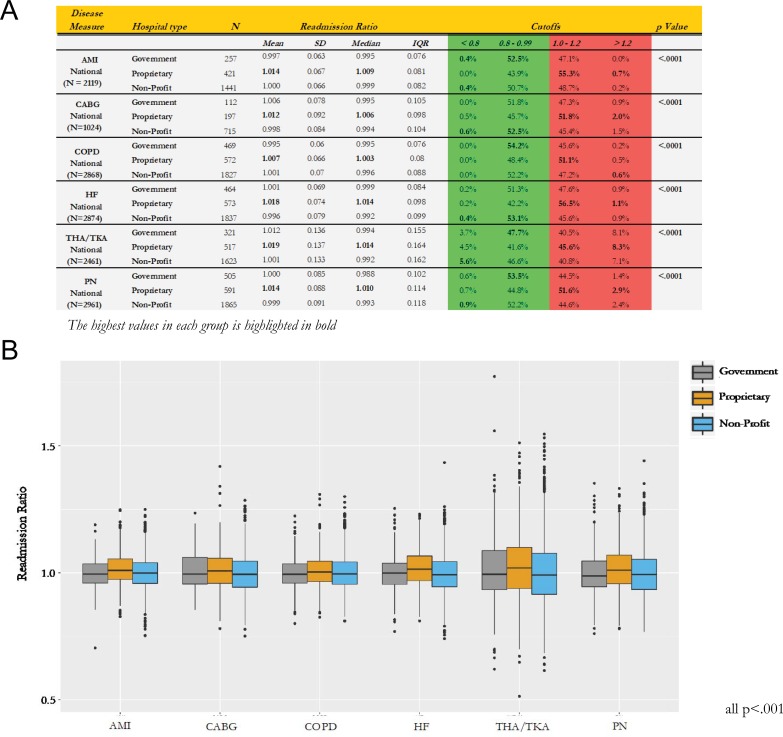
Readmission ratios for six major diseases: AMI, HF, CABG, PN, COPD, and THA/TKA by hospital type: Panel A reports the mean, median, standard deviation, as well as the results for the 4 intervals. Red zone represent readmission ration >1.0. The Green zone is readmission ratios < 1. Panel B reports the box and whisker plots of the results of the readmission ratios by hospital type and disease.

Data on readmission ratios from 14,307 disease specific hospital reports was obtained from the national Hospital Readmission Reduction Program (HRRP) from 2012–2015 for six major diseases: AMI, HF, CABG, PN, COPD, and THA/TKA (Data sets 2017). Hospital ownership type was sourced from a Center for Medicaid and Medicare Services report (i.e. government, proprietary or non-profit by Provider Identifier) [15]. Using the provider identifier in both HRRP and the ownership file, the two data sets were mapped together. The calculations of the excess readmission ratio are based on the methodology used for the calculation of the CMS 30-day risk standardized readmission measures for the Hospital Inpatient Quality Reporting Program as described in the instructions for HRRP database [15]. A readmission ratio less than 1 is considered good, where a ratio greater than one implies excess readmission controlled for disease severity. The readmission ratio was categorized into 4 intervals: <0.8, 0.8–0.99, 1.0–1.2 and >1.2 for each disease. Hospital ownership was additionally used to analyze the readmission ratios by focusing on the percentage of hospitals in each ratio category (Fig 1A). Readmission ratio of less <1 (Green Zone) was defined as a hospital having fewer readmissions than expected after adjustment for patient disease severity for the individual hospital. Hospitals with >1.0 (Red Zone) was defined as having more readmissions than expected. A Fischer exact analysis was conducted to calculate statistically significant differences between the groups of ownership and additionally the readmission ratios were graphed for each disease and ownership type by whisker-box plot. A list of the top and bottom 10 hospitals based on readmission ratio for all six major diseases was created. The readmission ratio of various hospitals according to the ownership was also mapped to their geographical location using Tableau 10.5.

## Results

**Fig 2:**
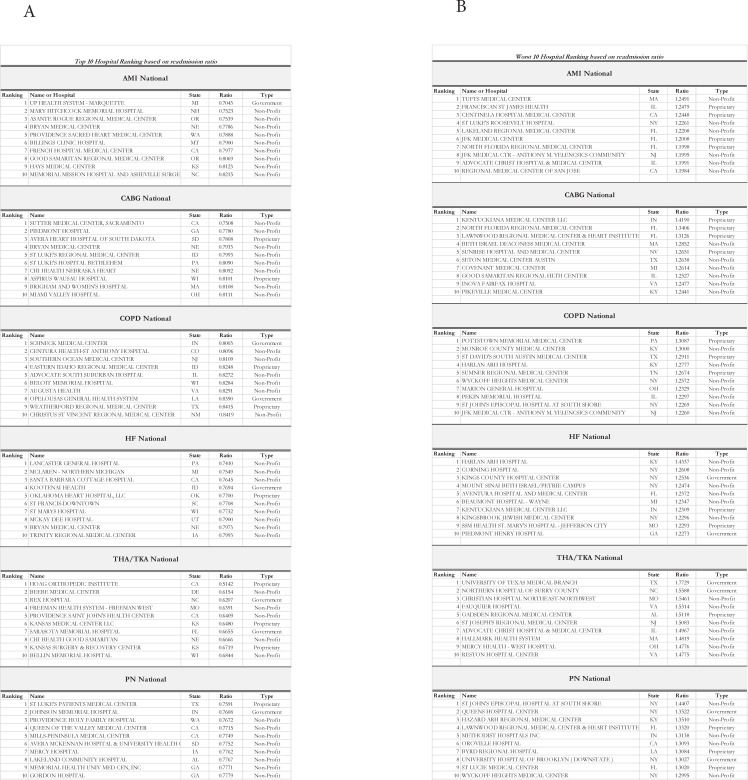
Panel A. A list of the top 10 best hospitals per disease readmission ratio for all six diseases. Panel B. A list of the bottom 10 hospitals per disease readmission ratio for all six diseases.

**Fig 3:**
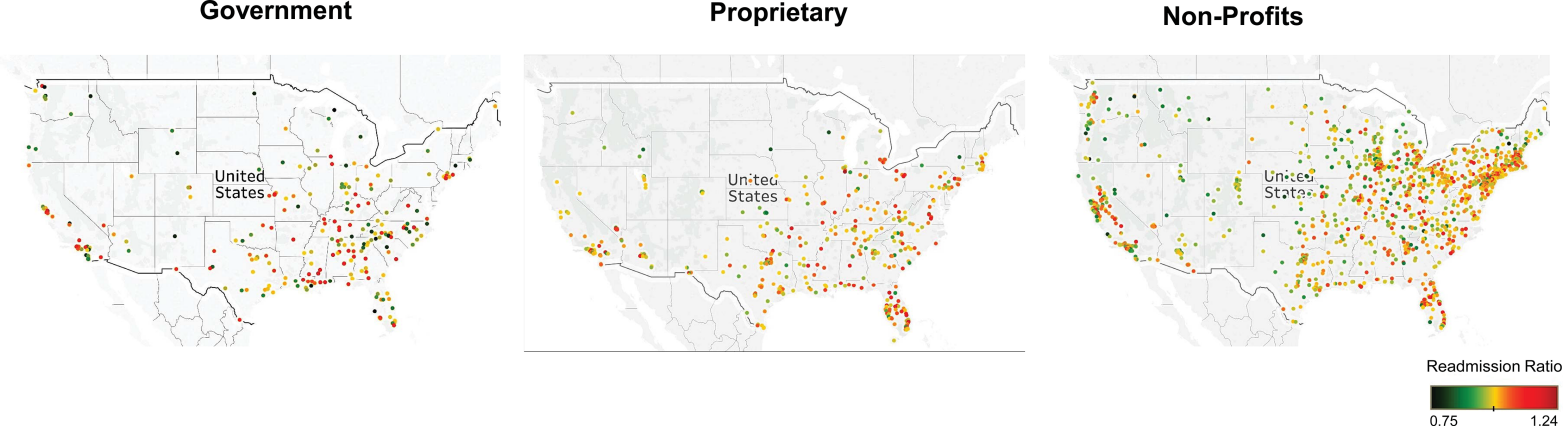
A geographical mapping of hospital readmission ratio results in three different panels by hospital type, Government, Proprietary, Non-Profit.

From the Fisher Exact analysis, we found a statistically significant difference between hospital ownership in readmission ratio among all six major diseases (all p < .0001, Fig 1A). The Green Zone (readmission ratio < 1) was predominated by government and non-profit hospitals compared to their proprietary counterparts (Fig 1A). In contrast, the Red Zone (readmission ratio > 1) was predominated by proprietary hospitals for all six major diseases (Fig 1A). As illustrated in Fig 1B, the median and mean readmission ratio was highest for proprietary hospitals for all six diseases (all p < .001). In the top 10 hospital readmission ratio rankings, the government and non-profit hospitals predominated the list for HF, AMI, COPD, and CABG while proprietary hospitals were only included in the topmost ranking only for THA/TKA and PN (Fig 2) (7 Government, 44 non-profits, 9 proprietary). When examining the bottom 10 hospital readmission ratios across all 6 diseases for 60 hospitals; proprietary hospital have a large percentage of membership in this list 28% versus 15%. The breakdown is 6 Government, 37 non-profits, 17 proprietary. We next examined the overall geographical distribution of government, non-profits, and for-profit hospitals based on their readmission score. All three categories of hospitals were uniformly distributed geographically across United States irrespective of their readmission ratio indicating that the differences in quality of care between government, non-profits, and for-profit hospitals does not relate to their geographical location (Fig 3).

## Discussion

The current study has identified that the government and non-profit hospitals have statistically significant lower readmission ratios compared to the proprietary hospitals for all six major diseases. With the rising cost of the healthcare services, an important priority before healthcare policy makers is how to reduce the cost of healthcare services without compromising the safety of patients. Readmissions cost the healthcare system monetary penalties, but a larger concern to patients are the missed days or weeks with family and friends when they would otherwise be outside of the hospital.

Multiple studies have shown that ownership structure of Health Care organizations (HCOs) affect the performance of providers and patient outcomes [7–14]. While hospitals are different than many other HCO’s, we compare the literature to other HCO as few studies on HCO ownership have been published. Similar to our study, Horwitz et al. have reported higher readmission rates for patients at for-profit hospitals among the 4474 hospitals analyzed for Medicare beneficiaries from July 2013-July 2014 [16]. Daras et al. have reported higher readmission rate for rehabilitation patients in the for-profit IRFs (Inpatient Rehabilitation Facilities) than the non-profit IRFs [8]. Although, the authors also reported geographical variation in their study with more readmission rates in IRFs in the South Atlantic and South Central regions than the New England, these readmissions were related to all cause-unplanned population which may have different mix of patients than what was observed in this study [8]. In another study, Devereaux et al. performed the meta-analysis of published and unpublished observational studies from 1973 to 1997, to compare the mortality rate of patients in for-profit vs not-for-profit dialysis centers, and reported significantly higher mortality risk associated with for-profit dialysis centers [9]. Another meta-analysis study based on 82 articles, performed by Comondore et al., from 1965–2003, reported higher quality of care in non-profit nursing homes compared to the for-profit facilities [10]. The non-profit nursing homes were associated with higher quality staffing and lower pressure ulcer prevalence compared to the for-profit nursing homes [10]. Hillmer et al. similarly reported better quality of care associated with non-profit nursing homes using qualitative systematic review of 38 studies from 1990–2002 [11]. Rosenau et al. reported that non-profits were judged 59% of the time superior, whereas for-profits were judged to be superior only 12% of the time [12]. Their study was based on the systematic review of two decades of articles published since 1980–2003 [12]. Some of the studies, however, have reported better care quality in for-profit institutions [13–14]. Leleu et al., for instance, have shown reduced readmission in for-profit teaching and fully integrated hospitals than their counterparts [13]. Akintoye et al., have reported reduced mortality in for-profit hospitals among HF patient from 2013–2014 nationwide [14]. There could be multiple reasons ascribed to this discrepancy such as specificity to a particular disease category, temporal differences or use of different database.

The analysis of readmission ratio reported in this study is based on the most current data set available yet since the start of the HRRP program in 2012. One additional caveat to readmission is a recently published study by Fonarow et al. showing that a reduction of readmission rate in HF patients, after HRRP implementation, was accompanied by a concomitant rise in the risk adjusted mortality rate [17]. Due to the nature of the government reported HRRP data, this new risk is unable to be assessed but requires further research in the future.

The Center for Healthcare Quality and Policy Reform (CHQPR) has suggested five basic approached to payment reform [18]: 1) Don’t pay providers for readmissions, 2) Provide incentive to providers to implement programs to reduce readmissions, 3) Pay providers bonuses/incentives based on readmission rates, 4) Don’t pay provider for readmissions meeting specific criteria, and 5) Make patient care comprehensive regardless of number of hospitalizations and readmissions. The larger concern from a policy perspective is why proprietary hospitals have such a different readmission ratio than their other counterparts. The relatively lack of resources at proprietary HCOs due to higher taxes and focus on maximizing the return on investment may lead to recruitment of less qualified staff or less investment in medical technology resulting in inferior quality of care than the government or non-profit institutions [4]. With the shrinking revenues in healthcare market and a move towards the value based payment system, the particular aspect of provider ownership on delivery of healthcare services is becoming more crucial. The policy makers need to carefully evaluate and design healthcare policies that mitigate potential risks associated with unintended revenue pressure in harming patients.

### Strengths and limitations

The major strengths of this study is that the findings described herein are based on nationally available HRRP database that includes all of the hospitals with the associated illness, currently operating across the United States. Due to the unrestricted availability of this publicly reported data, the results of the study can be independently investigated and reproduced by any research group working in this field. Another major strength of this study is that the HRRP database includes readmission ratio which is normalized based on the severity of illness of the patients treated in the hospital. The major limitations of this study is that the data are derived and limited by the accuracy of the disease severity adjustment. Another limitation is the accurate categorization of hospitals in different ownership types, this study is reliant on the proper categorization by the government. The final limitation in this study is that we only examine six disease categories collected by the government. The readmission ratio has not been calculated for all admissions to a hospital and different diseases could have different ratios.

## Conclusions

The underperformance of proprietary (for-profit) hospitals in the current study may be associated with several factors including stakeholder’s expectations for operating profits; different patient insurance portfolios; limitations of the HRRP disease adjustment; or other factors. As heath care markets continue becoming more consolidated, it is critical to conduct additional study to understand the impact of provider ownership on patient outcomes.
